# CAR-T Therapy in Relapsed Refractory Multiple Myeloma

**DOI:** 10.2174/0109298673268932230920063933

**Published:** 2023-09-27

**Authors:** Hong Ding, Yu Wu

**Affiliations:** 1 Department of Hematology, West China Hospital, Sichuan University, China

**Keywords:** Multiple myeloma, relapsed refractory multiple myeloma, immunotherapy, chimeric antigen receptor T-cells, bispecific antibodies, antibody-drug conjugates

## Abstract

Multiple myeloma is a plasma cell neoplasm. The emergence of proteasome inhibitors, immunomodulatory drugs, and anti-CD38 monoclonal antibodies has improved the prognosis of multiple myeloma patients. However, some patients are still insensitive to conventional therapy or frequently relapse after remission. Chemotherapy based on proteasome inhibitors or immunomodulatory drugs is ineffective in controlling the progression of relapsed refractory multiple myeloma. No consensus has been reached on treating relapsed refractory multiple myeloma to date. Recently chimeric antigen receptor T cells therapy has shown promising results that could achieve rapid remissions of patients and improve their prognoses. Additionally, most patients in chimeric antigen receptor T cell clinical trials were triple-refractory multiple myeloma patients, indicating that chimeric antigen receptor T cell immunotherapy could overcome drug resistance to new drugs. Since single immunotherapies are prone to acquired resistance, combination immunotherapies based on emerging immunotherapies may solve this issue. Achieving complete remission and minimal residual disease negative status as soon as possible is beneficial to patients. This paper reviewed the main chimeric antigen receptor T cell products in relapsed refractory multiple myeloma, and it explained the drug resistance mechanism and improvement methods of chimeric antigen receptor T cells therapy. This review summarized the best beneficiaries of chimeric antigen receptor T cell therapy and the salvage treatment of disease recurrence after chimeric antigen receptor T cell therapy, providing some ideas for the clinical application of chimeric antigen receptor T cells.

## 1 INTRODUCTION

Multiple myeloma (MM) is a highly heterogeneous plasma cell tumor with immune dysfunction [1, 2]. MM patients usually suffer from hypercalcemia, renal insufficiency, anemia, and bone disease [3]. With the advent of new therapeutic agents, such as proteasome inhibitors (PIs), immunomodulatory drugs (IMIDs), and anti-CD38 monoclonal antibodies, the overall survival of MM patients has significantly improved [4-6]. However, almost all patients with MM eventually relapse, especially those with high-risk genetic characteristics or extramedullary disease [7, 8]. As the disease progresses, plasma cells acquire different genetic abnormalities, causing the biological development of myeloma [9-11]. When plasma cells with specific genetic abnormalities reach the threshold, the cells gain clonal superiority and evolve branchingly, leading to drug resistance to conventional therapy [9-12].

MM patients reach relapsed/refractory status at different stages [13]. Most of these patients are primary refractory, and some relapse early or late after the first-line therapy bridges autologous stem cell transplantation [13]. The number of MM relapses decreases the medications a patient can consume. New emerging drugs are urgently needed to break the relapsed refractory multiple myeloma (RRMM) treatment bottleneck. In the last five years, emerging immunotherapies, such as chimeric antigen receptor T cells (CAR-T), bispecific antibodies (BsAbs), and antibody-drug conjugates (ADCs) have demonstrated promising results in RRMM patients [14-16]. CAR-T therapy has particularly exhibited a high response rate in RRMM, and it could also benefit patients with high-risk genetic characteristics or extramedullary diseases (EMD) [14, 15, 17]. The most widely used target of CAR-T therapy for MM is B cell maturity antigen (BCMA) [18, 19]. CAR-T products for MM are divided into BCMA-targeting CAR-T and non-BCMA-targeting CRA-T products. Food and Drug Administration (FDA) has approved two CAR-T drugs for MM so far, including idecabtagene vicleucel (ide-cel) and ciltacabtagene autoleucel (cilta-cel) [20, 21]. However, immunotherapies have drawbacks, such as antigen shedding, immune escape, and T-cell depletion [16, 19]. Researchers have attempted to overcome these issues in various ways, such as increasing the number of target antigens, optimizing the production process, and improving the structure of CAR-T products [16, 22].

This review summarizes the safety and efficacy data of the predominantly used CAR-T products targeting BCMA and nontargeting BCMA in RRMM. In addition, this paper introduces the mechanisms of resistance to CAR-T therapy and proposes some methods to improve efficacy and safety. Importantly, this review summarizes beneficiaries of CAR-T therapy in RRMM and provides advice on salvage therapy for disease recurrence following CAR-T treatment.

## 2 CAR-T THERAPY

In the past decade, CAR-T immunotherapy has achieved favorable results in other hematological diseases, such as diffuse large B-cell lymphoma (DLBCL) and acute lymphoblastic leukemia (ALL). Therefore, it is hypothesized to improve the outcome of RRMM patients as well [18, 23, 24].

CAR-T cells are genetically engineered T-cells *in vitro* that specifically recognize antigens on the surface of tumor cells through chimeric receptors, resulting in T cell activation and selective killing of tumor cells [19, 25]. CAR-T products comprise an antigen recognition domain, a hinge region, a transmembrane domain, and an intracellular signaling domain. The most common antigen recognition domain is the single-chain variable fragment (ScFv) [15].

According to different compositions of intracellular signal regions, CAR-T products can be divided into four generations [15, 26]. The first-generation chimeric antigen receptors (CARs) contain only CD3-zeta, which activates T-cells [15, 27]. The second-generation CARs increase CD28 or 4-1BB costimulatory domains, enhancing the function and persistence of CAR-T cells [26, 28]. The third-generation CARs comprise two or more costimulatory domains [14, 15]. The fourth-generation CARs increase suicide genes or key cytokines [15, 29]. Second-generation CAR-T products are currently utilized in most clinical trials [15]. CAR-T therapy has the advantage of directly recognizing tumor cells without relying on the major histocompatibility complex (MHC), making CAR-T suitable for any human leukocyte antigen (HLA)-type patient [30, 31]. However, CAR-T therapy can also cause adverse reactions, including cytokine-release syndrome (CRS), neurotoxicity, and hematological toxicity [32, 33]. Tolizumab and/or corticosteroids should be used when patients have Grade II or higher CRS [32, 34].

The target antigen of CARs needs to be significantly expressed in tumor cells, but it has low or no expression in normal cells [18]. The main target of CAR-T therapy in MM is BCMA (CD269/TNFRSF17), which belongs to the tumor necrosis factor (TNF) superfamily and is essential for plasma cell differentiation and long-term survival [18, 19]. BCMA is highly and preferentially expressed in malignant plasma cells but not in hematopoietic stem cells, T cells, immature B cells, and memory B cells [35, 36]. BCMA induces the PI3/AKT, MAPK, and NF-κB signaling cascades by binding with its ligands, a proliferation-inducing ligand (APRIL) and B-cell activating factor (BAFF), promoting tumor cell proliferation and survival [36, 37].

### 2.1 BCMA-directed CAR-T Products

CAR-T therapy is an emerging topic in MM immunotherapy. Ide-cel (bb2121) was the first CAR-T product approved by the FDA for MM, and it was the second-generation CAR-T directly targeting BCMA [20]. Several clinical trials have evaluated the efficacy and safety of ide-cel monotherapy or combination therapy. KarMMa (NCT03361748) included 140 RRMM patients with an ORR of 73% [20]. The CRB-401 study also achieved good results, with an ORR of 76% (Table **1**) [38].

Cilta-cel (LCAR B38M/JNJ 68284528) was the second CAR-T approved by the FDA for MM in February 2022 [15]. The single antigen recognition domain of cilta-cel contains two anti-BCMA ScFvs, which can remove MM cells with low BCMA expression and improve the affinity of CAR-T cells [15, 21]. In addition to the structure of the antigen recognition domain, there is also a difference in the target dose between cilta-cel and ide-cel. Cilta-cel has a small infusion dose and requires a longer time for *in vivo* expansion to have a significant effect [19]. In the CAR-TITUDE-1 (NCT03548207) study, 97 RRMM patients received cilta-cel infusion, and the ORR reached 97% [21]. The ORR of LEGEND-2 (NCT03090659) was 88% [39].

### 2.2 Non-BCMA-directed CAR-T Products

In addition to BCMA, researchers are exploring CAR-T therapy that targets other plasma cell markers. These markers include CD19, CD138, CD38, SLAM family member 7 (SLAMF7/CD139), and G protein-coupled receptor class C group 5 member D (GPRC5D).

CD19 is a surface marker of B cells but is not expressed in malignant plasma cells [40]. Anti-CD19 CAR-T therapy has shown favorable results in ALL and DLBCL [23, 41]. But, in terms of efficacy, the first anti-CD19 CAR-T clinical trial in MM failed, and 10 patients experienced disease progression [42]. Fortunately, CAR-T cells targeting BCMA and CD19 showed a higher ORR. The ORR of GC012F was 94.7% [43]. No patients had Grade IV/V CRS or neurotoxicity [43].

SLAMF7 is commonly expressed in immune cells but not in the nonhematopoietic system [44, 45]. FDA approved it in 2015 after the SLAMF7 monoclonal antibody (MoAb) elotuzumab combined with lenalidomide and dexamethasone significantly improved the prognosis of MM patients [46]. Single-target (NCT04499339, NCT03710421, and NCT04142619) and dual-target (NCT04662099) CAR-T products based on SLAMF7 are being explored by researchers.

CD138 is predominantly expressed in plasma and some epithelial cells [18, 47]. In 2015, the first anti-CD138 CAR-T clinical trial showed poor efficacy, with an ORR of 80%, because 80% of patients were in a stable disease stage [48]. Currently, anti-CD138 single-target (NCT03672318) and multitarget CAR-T (NCT03778346) products are being examined.

CD38 is widely expressed in immune cells and normal tissues [57-59]. The CD38 MoAbs daratumumab and isatuximab demonstrated favorable results in RRMM patients [60-62]. Therefore, anti-CD38 single-target (NCT03464916) and multitarget CAR-T (NCT03767751, NCT03638206, and NCT03271632) products are being developed. Anti-CD38 CAR-T cells showed the side effects of extensively killing immune cells and off-target effects. Consequently, researchers incorporated suicide genes into the costimulatory domain to eliminate cytotoxicity (NCT03464916) [59, 63].

GPRC5D is a G protein-coupled receptor with an expression distribution similar to BCMA and is a potential target of CAR-T in MM [64-66]. Early clinical experiments showed that anti-GPRC5D CAR-T influenced the BCMA escape model [67]. In MCARH109, a Phase-I clinical trial targeting GPRC5D, 17 RRMM patients achieved a 71% ORR [68]. Of these, 10 patients had previously received BCMA-directed treatment; the ORR was 70% in this group [68]. Another GPRC5D-targeted clinical trial, OriCAR-017, included 10 RRMM patients with an ORR of 100%; five patients resisted anti-BCMA CAR-T products [69]. In addition, a multicenter clinical trial targeting GPRC5D updated the experimental data of Section Part A at the 2022 American Society of Hematology (ASH) conference [70]. The CC-95266-MM-001 study included 17 RRMM patients who were resistant to at least three lines of therapy and reached an ORR of 86% [70]. Seven patients in this study had received anti-BCMA treatment before the study, and four patients achieved clinical responses [70]. Other anti-GPRC5D CAR-T clinical trials are ongoing, including NCT05219721 and NCT05016778. Despite positive results, the studies on anti-GPRC5D CAR-T therapy had a short follow-up period and a small sample size; therefore, additional study data are needed to confirm the efficacy and safety of anti-GPRC5D CAR-T therapy.

## 3 RESISTANCE MECHANISM OF CAR-T THERAPY

**Fig. (1):**
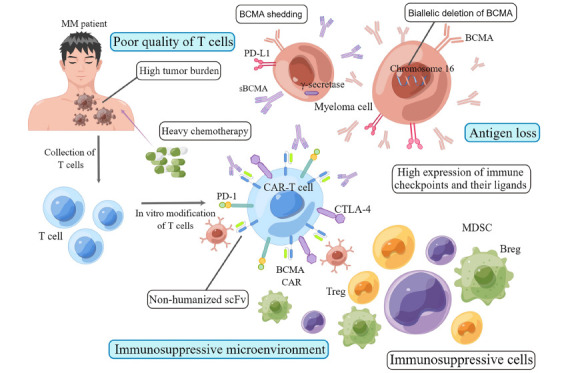
Resistance mechanism of CAR-T therapy. (**1**) Antigen loss. Soluble BCMA, which is generated from the combination of BCMA and γ-secretase, can interact with CAR-T ScFv, leading to antigen hiding of CAR-T. (**2**) Poor quality of T cells. The content of memory T cells was lower in patients with high tumor burden and heavy chemotherapy. Non-humanized ScFv may produce humoral immunogenicity and reduce the persistence of CAR-T. (**3**) Immunosuppressive microenvironment. Several kinds of immuno- suppressive cells in the MM microenvironment inhibit the effect of CAR-T in various ways. (By Figdraw). **Abbreviations:** CAR-T, chimeric antigen receptor T cells; BCMA, B-cell maturity antigen; sBCMA, soluble BCMA; PD-L1, programmed death-ligand 1; PD-1, programmed death-1; scFv, single-chain variable fragment; CTLA-4, cytotoxic T-lymphocyte-associated antigen-4; MDSC, myeloid-derived suppressor cells; Treg, regulatory T-cells; Breg, regulatory B-cells.

Although the response rate of CAR-T products targeting BCMA is high, patients still have the risk of recurrence due to antigen loss, poor T-cell quality, and an immunosuppressive microenvironment (Fig.**1**) [16, 19, 71]. First, antigen loss is an important resistance mechanism of CAR-T therapy [72]. BCMA is the primary target of CAR-T products in MM, and its low expression may be associated with poor efficacy [73]. Moreover, BCMA falls off the surface of plasma cells and releases soluble BCMA in abundance by binding with γ-secretase [74-76]. These soluble antigens can bind to CAR-T ScFv, resulting in antigen hiding and efficacy and duration reduction of CAR-T [74-76]. After CAR-T therapy targeting BCMA, the BCMA expression on the surface of MM cells decreases significantly, which may lead to immune escape [77, 78].

Second, the quality of autologous T cells also affects the efficacy of CAR-T cells [16]. Patients in the early stage of MM who have not received heavy induction therapy have a higher proportion of memory T cells and CD4/CD8 T cells; therefore, CAR-T cells can achieve better proliferation and persistence [79-82]. Progressive dysfunction of T-cells occurs during MM progression [83, 84]. Studies have shown that senescent T-cell subsets appear in the precancerous state of MM [1, 2, 25]. In the later stage of MM, the programmed cell death protein-1 (PD-1) and cytotoxic T-lymphocyte-associated antigen-4 (CTLA-4) expressions on the T-cell surface increase, and the quantity and quality of T-cells decrease [1, 2, 25]. Besides, non-humanized ScFv may produce humoral immunogenicity to reduce the persistence of CAR-T and cause recurrence after CAR-T therapy. For MM patients with a high tumor burden, CAR-T cells deplete faster, limiting the efficacy and causing disease relapse [20, 85].

Third, the immunosuppressive microenvironment can also decrease the efficacy of CAR-T cells. Several kinds of immunosuppressive cells in the microenvironment of MM inhibit the effect of immune cells in various ways [86]. These cells include myeloid-derived suppressor cells (MDSCs), regulatory T-cells (Tregs), and regulatory B-cells (Bregs) [86]. Myeloid cells and their precursors in bone marrow can be transformed into MDSCs [87]. MDSCs produce reactive oxygen and nitrogen species during amino acid consumption, which cause oxidative stress and inhibit the immune function of T-cells [86]. MDSCs and effector T-cells can induce Tregs [86, 87]. Tregs secrete the inhibitory cytokines interleukin-10 (IL-10) and transforming growth factor beta (TGF-β) to suppress the functions of natural killer cells (NK-cells) and B-cells [86]. Moreover, the immune checkpoints CTLA-4 and lymphocyte-activation gene 3 (LAG3) on the Tregs surface combine with their ligands to limit the function of dendritic cells (DCs) [86]. In addition, Tregs cells may also induce DC cells to express the tryptophan metabolism catalytic enzyme indoleamine 2,3-dioxygenase (IDO) to inhibit the immune effect of DCs [88]. Bregs also secrete the inhibitory cytokines IL-10, IL-35, and TGF- β to inhibit T cells [89]. With the progression of MM, the immune checkpoint expression of T cells increases, weakening the activity of CAR-T cells [78, 90]. In addition, some MM tumor cells can highly express immune checkpoint ligands, such as programmed cell death ligand-1 (PD-L1), and escape immune surveillance by combining immune checkpoints on the surface of immune cells [91, 92].

## 4 POTENTIAL STRATEGIES TO OVERCOME RESISTANCE TO CAR-T THERAPY

**Fig. (2):**
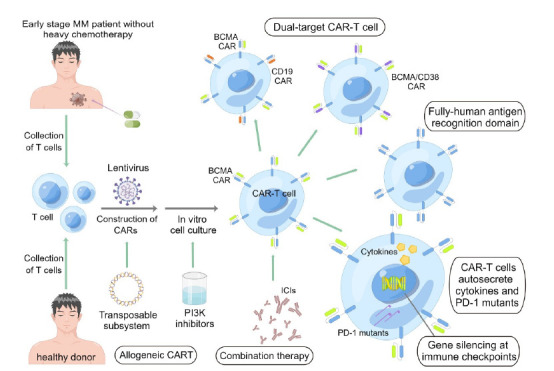
Potential strategies for overcoming resistance in CAR-T. (**1**) Optimizing the CAR-T structure. Dual-target CAR-T products are constructed by transferring two CAR structures targeting different antigens in the same CAR-T cell or constructing two different antigen recognition domains in the same CAR structure. (**2**) Reducing the immunogenicity of antigens. Humanized ScFv and CARS based on transposable subsystems can reduce immunogenicity. (**3**) Improving the quality of T cells. Autologous T cells should be collected and preserved as early as possible after the patient has reached remission and before heavy chemotherapy. The proliferation of T cells in healthy donors is higher than in patients; therefore, allogenic CAR-T therapy can also be selected. In the process of T-cell culture *in vitro*, PI3K inhibitors are added to increase the content of memory T cells. CAR-T cells auto-secrete important cytokines to promote the killing of tumor cells. (**4**) Changing the immunosuppressive microenvironment. The combination of ICIs and CAR-T improves the antitumor effect. New gene editing techniques can reduce immunosuppression, including gene silencing at immune checkpoints or introducing PD-1 mutant genes with high affinity. (By Figdraw). **Abbreviations:** CAR-T, chimeric antigen receptor T cells; BCMA, B-cell maturity antigen; PD-1, programmed death-1; PI3K, phosphatidylinositol-3 kinase; ICIs, immune checkpoint inhibitors.

Considering the above factors that limit the efficacy of CAR-T, researchers have proposed several solutions, including optimizing the CAR-T structure, reducing antigens’ immunogenicity, improving T cells’ quality, and changing the immunosuppressive microenvironment to overcome this issue (Fig. **2**).

### 4.1 Optimizing the CAR-T Structure

Researchers are developing dual-target or multitarget CAR-T products to improve efficacy and reduce the risk of negative recurrence. These products include transferring two CAR structures targeting different antigens in the same CAR-T cell or constructing two tandem different antigen recognition domains in the same CAR structure [93, 94]. BCMA, CD19, CD38, and SLAMF7 are the primary targets of dual-target CAR-T products in MM. The reaction rate of dual-target CAR-T and the rate of side effects are higher and lower than single-target CAR-T, respectively. GC012F (NCT04236011, NCT04182581) is a bispecific CAR-T product targeting BCMA and CD19 currently in early Phase I and has an ORR of 94.7% [43]. Only two patients developed Grade III or higher CRS, and none had neurotoxicity [43]. BM38 is a humanized bispecific CAR-T cell product targeting BCMA and CD38; the ORR of the BM38 CAR (ChiCTR1800018143) trial was 87% [49]. In this study, 22% of the patients developed Grade III or higher CRS and no neurotoxicity [49]. Moreover, CAR-T clinical trials targeting BCMA and SLAMF are also recruiting (NCT04662099, NCT04156269, and NCT03196414).

In addition to using bispecific CAR-T cells, patients can achieve multitarget therapy by sequential infusion of various CAR-T products. The Affiliated Hospital of Xuzhou Medical University reported the results of sequential infusion of anti-CD19 and anti-BCMA CAR-T (ChiCTR-OIC-17011272) in 2019 [50]. The study’s ORR was 95%, and only one person developed Grade III CRS [50]. Recently, a study at Suzhou University reported the results of an exploratory trial of anti-CD19 and anti-BCMA CAR-T sequential therapy (NCT03455972) for high-risk NDMM patients [51]. The ORR was 100%, and no patient had nervous system toxicity or Grade III or higher CRS [51].

### 4.2 Reducing the Immunogenicity of Antigens

Several anti-BCMA CAR-T products containing humanized ScFv developed to reduce antigen immunogenicity include orvacabtagene autoleucel, CT053, MCARH171, CT103A, and FHVH33 [95-97]. Lummicar-1 (China) and Lummicar-2 (USA) trials evaluated the safety and tolerance of CT053. Lummicar-1 (NCT03975907) included 14 patients with RRMM, with a 100% ORR, and no patients developed CRS grade ≥ 3 or neurotoxicity [52]. Lummicar-2 (NCT03915184) had 100% ORR, and no patient had CRS grade ≥ 3 or neurotoxicity [53]. High-level adverse effects (AEs) of humanized CAR-T were significantly lower than in other CAR-T products.

A previous study shows that CARs constructed by the transposable subsystems benefit the development and proliferation of memory stem cells more than CARs transduced by lentivirus [98]. Moreover, CARs based on transposable subsystems can reduce immunogenicity [19]. P-BCMA-101 is a representative drug, and a Phase I/II clinical trial (NCT03288493) showed that its ORR reached 55%. Only one patient had CRS Grade III or higher [54]. In addition, the peak time and duration of P-BCMA-101 were significantly prolonged to two to three weeks and 1.5 years, respectively [54].

### 4.3 Improving the Quality of T Cells

Naïve T cells and memory T cells have better proliferation and slower aging processes [99]. Researchers have tried to increase the number of memory T cells using various techniques to improve the persistence of CAR-T. Bb21217, which has the same CAR structure as bb2121, improves the production process of T cells [100]. When T cells were cultured *in vitro*, the PI3K inhibitor, bb007, was added to enrich memory-like T cells, improving the proliferation of CAR-T cells [55]. The early results of the CRB-402 (NCT03274219) trial showed a similar response rate of bb21217 to bb2121 [55]. Long-term follow-up is required to show differences in persistence.

Some cytokines, such as IL-12, IL-18, IL-15, IL-21, and IL-23, can promote T cells’ expansion and immune function [19, 101-103]. For example, IL-12 has an antitumor activity that can overcome the immunosuppressive microenvironment and induce innate and acquired immunity of effector cells [49]. The fourth-generation CAR-T integrates essential cytokine genes in the costimulatory domain, which can activate T cells and secrete cytokines to promote the killing effect on tumor cells [19]. Recently, researchers at Southern Medical University constructed an anti-BCMA CAR-T product that secreted IL-7 and CCL19; initial clinical trials (NCT03778346) showed safety and efficacy [101].

The quality of T cells decreases with the progression of the disease and the superposition of chemotherapy. Therefore, patients should collect and preserve T cells as soon as possible after remission to improve the proliferation and persistence of CAR-T cells [104, 105]. Autologous CAR-T therapy demonstrated favorable results in MM; however, its drawback is that approximately one month is required to prepare CAR-T products [19]. Some patients cannot wait this long because of rapid disease progression; thus, allogeneic CAR-T can be collected from healthy people because the quality of T cells from healthy individuals is better than those from MM patients [19]. The limitations are the graft-versus-host disease (GvHD) and the short duration of CAR-T cells [14]. The first allogeneic BCMA CAR-T product was allo-715 [56]. Researchers utilized gene editing techniques to knock out the T-cell receptor alpha constant (TRAC) and CD52 gene to reduce the incidence of GvHD [56]. In addition, an anti-CD52 monoclonal antibody, allo-647, was used to consume lymphocytes [56]. The ORR of the DL3 and DL4 groups in the UNIVERSAL trial (NCT04093596) was 61.5% [56].

### 4.4 Changing the Immunosuppressive Microenvironment

Lymphocyte depletion can eliminate immunosuppressive cells in the microenvironment through fludarabine and cyclophosphamide as the preferred drugs [67, 106]. Hematopoietic stem cell transplantation provides a favorable immune environment for T cell recolonization [106]. Normally, immune checkpoints of T cells prevent killing self-cells such as PD-1 and CTLA-4 [107, 108]. In the late stage of MM, the expression level of immune checkpoint molecules is significantly upregulated [1, 2, 25]. Tumor cells evade the surveillance and attack of the immune system by overexpressing immune checkpoint ligands, such as PD-L1 [92]. Studies have found that immune checkpoint inhibitors (ICIs) combined with CAR-T can improve the antitumor effect. A study showed a synergistic effect between PD-1 MoAb and CAR-T therapy targeting BCMA and SLAMF7 [90]. A Phase-II clinical trial of the fourth-generation CAR-T targeting BCMA and secreting PD-1 mutants, which have a high affinity for PD-L1, is also underway (NCT04162119). In addition, gene silencing and functional inhibition at immune checkpoints can also improve the efficacy of antitumor therapy [109]. However, the complete knockout of PD-1 in CAR-T cells enhances cytotoxicity and shortens the duration of CAR-T cells [110, 111]. Exploring the optimal threshold of gene editing knockdown of immune checkpoints or the best target for silencing immune checkpoints is necessary. Clinical trials of knocking out multiple immune checkpoints in CAR-T therapy are currently being explored [112, 113].

## 5 BEST BENEFICIARIES OF CAR-T THERAPY IN RRMM PATIENTS

The biological characteristics of the disease and structural features of CAR-T products primarily affect the efficacy of CAR-T. The biological characteristics of MM include genetic characteristics, extramedullary lesions, previous treatment, tumor burden, and the type of disease [7, 114]. High-risk chromosome abnormalities are independent prognostic factors for MM. These include t (4; 14), t (14; 16), t (14; 20), del (17p), and + 1q, which are associated with adverse outcomes, especially poor PFS [7, 114-117]. The extramedullary disease is also significantly associated with the poor prognosis of RRMM patients treated with CAR-T [7]. The extramedullary disease is an independent predictor of disease progression and an independent prognostic factor for disease recurrence [7]. T cells progressively fail in the disease progression, failing more abundantly in patients who have received heavy treatment (≥ three lines) [104, 105]. Therefore, the quality of CAR-T cells is significantly reduced, causing cells to be ineffective or non-durable. Exposure to multiple therapies (≥ three lines) before CAR-T therapy is an independent predictor of PFS [7]. The disease type of MM is also related to the prognosis of patients. MM patients with simple light chains are likelier to suffer kidney damage and have poorer prognoses than those with IgA and IgG immunoglobulins [7]. A high tumor burden at baseline also significantly reduces the efficacy of CAR-T cells and leads to T-cell exhaustion [114].

CAR-T products’ structure and infusion dose correlate with efficacy [8]. CAR-T can be divided into single and dual epitope-binding products according to the number of binding domains on the same T cell. Most single-chain variable regions (ScFvs) in CAR-T products are derived from humans and mice. The double epitope-binding products have higher ORR than the single epitope-binding products, and the human products are better than the murine products [8]. A study showed that BCMA-targeted CAR-T products were more effective than non-BCMA-targeted CAR-T products [118]. In addition, higher dose levels of CAR-T cell infusion are associated with higher ORR in RRMM [8, 119].

A meta-analysis involving 681 patients showed that the high-dose CAR-T cell group (median dose of CAR-T cells infusion > 100 × 10^6^), the young groups (< 60 years), and the few prior treatment groups (< six lines) had higher ORRs than other subgroups [8]. Moreover, high-grade CRS was more common in the high-dose and young groups [8]. MM patients with and without the extramedullary disease had similar ORRs, but PFS in the non-EMD group was significantly longer than in the EMD group [8]. In addition, the expression level of BCMA at baseline had an insignificant effect on ORR [8]. Contrary to other studies, this meta-analysis found no significant difference in ORR and PFS between patients with high and low genetic risks [8].

Based on current data, the present study hypothesizes that young MM patients (< 60 years) with less prior treatment (≥ three lines), no extramedullary disease, and low-risk cytogenetic features respond better to CAR-T therapy than other patients. In addition, dual epitope-binding CAR-T products and humanized CAR-T products have the optimal effect and the best safety, respectively [8]. More clinical trials and extensive study populations are needed to identify the best beneficiaries of CAR-T therapy.

## 6 SALVAGE THERAPIES OF RELAPSE IN RRMM PATIENTS

The response rate of CAR-T treatment is high. However, most MM patients relapse, especially those with high-risk genetic characteristics and extramedullary disease [119, 120]. For MM patients who receive anti-BCMA CAR-T for the first time, CAR-T cell reinfusion can be performed based on the expression of BCMA antigen when the patients do not respond to CAR-T or relapse after remission [120-122]. If the BCMA antigen’s expression is still high, patients can be re-infused with single- or dual-BCMA-targeted CAR-T products. Optional dual-target CAR-T products include BCMA/CD19, BCMA/CD38, BCMA/SLAMF7, BCMA/TACI, and BCMA/GPRC5D CAR-T [14, 43, 123, 124]. If patients relapse with BCMA negative status, CAR-T products targeting other antigens, such as those targeting Fc receptor homolog 5 (FcRH5) or GPRC5D, can be selected [15]. When the expression of BCMA is low, patients can choose CAR-NK cell therapy as the alternative to CAR-T therapy [125].

Preclinical studies preliminarily verified the efficacy and safety of SLAMF7-CAR NK cells, natural killer group 2 member D (NKG2D)-CAR NK cells, and BCMA-CAR NK cells in MM [19, 126, 127]. Moreover, BCMA CAR NK-92 cells developed by Suzhou Asclepius Company used in RRMM patients (NCT03940833) demonstrated that the safety of CAR-NK cells was high, and CRS or neurotoxicity occurred in few patients [19]. Patients with recurrence after anti-BCMA CAR-T therapy can choose additional salvage treatments, including BsAbs, ADCs, hematopoietic stem cell transplantation, treatment based on new small molecule inhibitors (for example, selinexor and venetoclax), and doublet/triplet/quadruplet combination therapy [120].

Recently, Blood published the results of a retrospective study on the outcomes of different salvage treatments (≥ one line) in 79 MM patients with progression after anti-BCMA CAR-T therapy [120]. The study found that T cell-engaging therapies, including CAR-T and BsAbs, had the most significant effect, producing a high response rate and lasting response [120]. The median follow-up time was more than 21 months, the ORR was 91.4%, and the median OS was unreachable [120]. Hematopoietic stem cell transplantation is another potential salvage therapy. The ORR was 71.4% and 100% for autologous stem cell transplantation (ASCT) and allogeneic stem cell transplantation (allo-SCT), respectively [120]. The median OS was 23.2 months [120]. Most patients chose multidrug combination therapy or treatment based on new small molecular inhibitors because they had at least three lines of drug resistance before receiving the first CAR-T treatment; however, the response rates were low [120]. The choice of follow-up treatment for recurrence after CAR-T may be affected by factors such as the biological characteristics of patients, time of recurrence, doctor’s preference, accessibility, and quality of new drugs. Prospective studies are needed to develop optimal salvage treatment strategies.

## 7 FUTURE PROSPECTS

The advancement of CAR-T technology in recent years has been rapid, leading to enhanced safety and efficacy of CAR-T. The effectiveness of CAR-T cells is primarily influenced by factors such as antigen escape, T-cell exhaustion, and immunosuppressive microenvironment [16, 19, 71]. To address the issue of antigen escape, scholars have directed their attention towards the development of non-BCMA-directed CAR-T and multi-target CAR-T therapies, alongside augmenting the density of target antigens on malignant cells [49, 50, 128-133]. Furthermore, the effectiveness of CAR-T is impacted by T-cell exhaustion. In addition to optimizing the costimulatory domain and source of CAR-T cells, there exist alternative approaches to increase T-cell functionality and longevity. These include the inhibition of signaling pathways associated with T-cell exhaustion and the enhancement of CAR-T cells' anti-tumor capabilities [43, 49, 50, 52, 53, 93, 94, 134-137]. PIK3/AKT pathway regulates T-cell proliferation and differentiation [138]. A recent study showed that dasatinib, an inhibitor of PIK3, could reverse T-cell exhaustion by downregulating exhaustion-related gene expression [138, 139]. T cells redirected for universal cytokine-mediated killing (TRUCKs), a type of next-generation CAR-T, enhance the expression of immune-stimulatory receptors within CAR-T cells *via* gene editing techniques. This augmentation facilitates the secretion of cytokines from CAR-T cells, enhancing their anti-tumor efficacy and inducing alterations in the tumor microenvironment [138, 140]. The researchers have demonstrated that the removal of the gene responsible for the impairment of CAR-T cells results in an extended duration of their presence [138, 140]. Additionally, some progress has been achieved in addressing the challenges posed by immunosuppressive microenvironments. The negative regulation of T cell activation through the PD-1/PD-L1 pathway can be overcome by inhibiting this pathway [141]. Currently, there exist three methods for interfering with PD-1/PD-L1 signaling. Firstly, researchers employ the fusion of extracellular PD-1 with the CD28 costimulatory domain of CAR-T cells [142]. This fusion enables the conversion of the immunosuppressive signal into an immunoactivating signal, thereby augmenting the efficacy of CAR-T cells [142]. Secondly, through gene editing, CAR-T cells are engineered to secrete PD-1 Fc fusion protein, which binds to PD-L1 and hinders its inhibition of T cells [143]. Finally, the researchers employed shRNA to introduce CAR-T cells with the aim of suppressing PD-1 expression, mitigating the inhibitory impact of PD-1/PD-L1 on T cells [144]. In comparison to conventional CAR-T cells, these newly developed CAR-T cells demonstrated superior efficacy in a limited patient cohort [143, 144]. To comprehensively evaluate the feasibility and effectiveness, it is imperative to expand the study population (Table **2**).

Neurotoxicity and CRS are common adverse effects of CAR-T therapy [32, 33, 140]. Cell surface antigens or intracellular factors can be incorporated into CAR-T cells through gene transduction [145]. This integration of genes leads to the expression of specific substances, rendering CAR-T cells responsive to them. Consequently, these substances can be eliminated through mechanisms such as ADCC, CDC, or apoptosis [145].

In terms of accessibility, the high price and complicated delivery process have affected the popularity of CAR-T [146]. In RRMM, CAR-T therapy has good efficacy, but it is expensive. Two CAR-T products have been approved by the FDA for RRMM: ide-cel and cilta -cell [15, 20]. The monthly price in the United States is $442,705 and $465,050, including hospitalization, medication and non-medication [147]. CT103A was officially approved in China on June 30^th^, 2023, while the price has not been disclosed yet. Despite the high cost of commercial CAR-T, an increasing number of clinical trials on RRMM are enrolling. Consequently, individuals with limited financial means can avail themselves of CAR-T treatment through clinical trials. Traditional CAR-T, primarily sourced from MM patients, have minor drawbacks including long manufacturing time and indeterminate T cell quantity and quality [138, 143]. The utilization of gene editing technology allows for the transformation of T cells acquired from healthy donors, developing allogeneic CAR-T cells and augmenting the availability of CAR-T therapy [138, 143].

## CONCLUSION

The outcome of MM patients has been significantly improved with the development of new drugs, but nearly all MM patients eventually relapse. Therefore, a new treatment for MM patients is crucially needed [4-8]. Young MM patients (< 60 years) who have not received much treatment previously (≥ three lines) and do not have high-risk genetic characteristics (t (4; 14), t (14; 16), t (14; 20), del (17p), +1q and p53 mutation) or extramedullary diseases would benefit most from CAR-T therapy [7, 8]. RRMM patients with high-risk genetic abnormalities or extramedullary diseases have a high response rate with CAR-T treatment; however, whether their survival can be significantly improved is unclear with the current data [8, 105, 115, 119]. In addition, BCMA expression levels’ effect on CAR-T efficacy in MM is ambiguous [8, 73, 114]. Research on more clinical data is needed to confirm these hypotheses.

The structural characteristics of CAR-T products also affect the efficacy of CAR-T. Humanized and double-epitope CAR-T products offer efficacy and safety [8]. A higher infusion dose of CAR-T cells causes a higher response rate and increases the incidence of CRS and neurotoxicity [7, 8, 119]. Several CAR-T clinical trials with different targets, dosages, and therapeutic strategies in MM are ongoing. CAR-T therapy is expected to be included in the guidelines to improve survival outcomes in MM patients.

In the emerging immunotherapy of MM, research regarding combining it with existing treatments to maximize the benefits for the survival of MM patients, especially RRMM patients, is challenging. More clinical trials are required to incorporate emerging immunotherapy into induction and consolidation therapies separately or jointly to explore the best treatment combination mode, time node, and order of use. Based on the current research, this study suggests that RRMM patients with no economic pressure should receive emerging immunotherapies as early as possible when the disease burden is low. If the single immunotherapy combined with chemotherapy is ineffective, other immunotherapies or targeted therapies should be combined to assist the patient in achieving complete remission quickly. When the patients reach and maintain an MRD-negative status as soon as possible, their survival time may be longer. The Nordic Myeloma Research Group found that the duration of response to the first-line therapy affected the prognosis for RRMM the most [148]. In the early stage of MM, the immune system function of patients has not been destroyed completely; thus, immunotherapies have a better effect theoretically. In contrast, patients without heavy chemotherapy have a higher proportion of memory T cells and CD4/CD8 T cells [79, 80]. Autologous CAR-T cells can achieve better proliferation and persistence [81, 82]. Younger patients are more tolerant of immunotherapy and recover quickly, even if they encounter significant AEs. In addition, immunotherapy combined with chemotherapy assists patients in reaching MRD-negative status as soon as possible, which is closely related to the prognosis of patients [149, 150].

The recurrence of MM after CAR-T treatment is also challenging. Different treatment options depend on BCMA expression levels [120-122]. If BCMA is still expressed, reinfusing anti-BCMA CAR-T products or dual-target CAR-T products containing BCMA is possible [14, 43, 123, 124]. Patients with low or no BCMA expression could choose other emerging immunotherapies, including CAR-NK therapy, BsAbs, or ADCs [120, 125]. If a patient’s financial or medical condition is unavailable, the individual could choose other salvage treatments such as hematopoietic stem cell transplantation, treatment based on novel small-molecule inhibitors (for example, selinexor and venetoclax), and multidrug combination therapy [120]. It is important to note that we should avoid ICIs in combination because of safety risks. Immune checkpoint inhibitors are effective in other hematological tumors, but the risks outweigh the benefits in MM [151, 152]. The possible reason is that progressive T-cell dysfunction occurs in the progression of MM, so it is difficult to reactivate T cells [1, 153, 154]. Another reason may be that many immunosuppressive cell subsets are located in the tumor microenvironment and inhibit the function of cytotoxic T cells [154, 155].

In summary, when compared to conventional chemotherapy medications and emerging drugs such as PIs and IMIDs, CAR-T has demonstrated superior effectiveness in RRMM, even among patients with high-risk genetic profiles and extramedullary disease. Currently, CAR-T therapy has not been incorporated into established guidelines, necessitating further investigation into the optimal timing, administration method, and combination treatment approach for CAR-T. Additionally, the issue of CAR-T resistance poses a significant challenge, as the efficacy of CAR-T is limited by antigen escape, T-cell exhaustion, and immunosuppression microenvironment. Scholars are currently investigating the advancement of CAR-T products in order to address the issue of CAR-T resistance. The primary approaches involve avoiding antigen escape, overcoming T-cell exhaustion, and improving the immunosuppressive microenvironment. Salvage therapy after CAR-T treatment remains uncertain. It has been proposed that the selection of treatment options could be guided by the level of BCMA expression. MM patients may benefit from CAR-T treatment, but further data is required to promote the utilization and popularity of CAR-T therapy.

## Figures and Tables

**Table 1:** Major clinical trials of emerging therapies.

**Name**	**Trail ID**	**Vector**	**Population**	**Target**	**Single or Combined Treatment**	**Status**	**Efficiency**	**Safety**
Ide-cel	NCT03361748 (KarMMa) [20]	Lentivirus	RRMM (≥3 lines, including anti-CD38 MoAb, n=128)	BCMA	Single	Phase 2 (active, not recruiting)	ORR 73%; ≥ CR 33%; MRD^-^ rate 26%; median PFS 8.8 months	CRS 84%, ≥ 3 grade 5%; neurotoxicity 18%, ≥ 3 grade 3%
Ide-cel	NCT02658929 (CRB-401) [38]	Lentivirus	RRMM (≥3 lines, including anti-CD38 MoAb, n=62)	BCMA	Single	Phase 1 (active, not recruiting)	ORR 76%; ≥ CR 39%; MRD^-^ rate 48%; median PFS 8.8 months	CRS 76%, ≥ 3 grade 7%; neurotoxicity 44%, ≥ 3 grade 3%
Cilta-cel	NCT03548207 (CAR-TITUDE-1) [21]	Lentivirus	RRMM (≥3 lines, including anti-CD38 MoAb, n=97)	BCMA	Single	Phase 1b/2 (completed)	ORR 97%; sCR 67%; MRD^-^ rate 93%; median PFS not reached	CRS 95%, ≥ 3 grade 4%; neurotoxicity 21%, ≥ 3 grade 9%
Cilta-cel /LCAR-B38M	NCT03090659 (LEGEND-2) [39]	Lentivirus	RRMM (≥3 lines, including anti-CD38 MoAb, n=57)	BCMA	Single	Phase 1/2 (active, not recruiting)	ORR 88%; CR 68%; MRD^-^ rate 63%; median PFS 15 months	CRS 90%, ≥ 3 grade 7%; neurotoxicity 2%, ≥ 3 grade 0%
GC012F	NCT04236011, NCT04182581 [43]	Lentivirus	RRMM (≥2 lines, including anti-CD38 MoAb, n=19)	BCMA/CD19	Single	Early phase 1 (recruiting)	ORR 95%; sCR 84%; MRD^-^ rate 84%	CRS 95%, ≥ 3 grade 11%; neurotoxicity 0%
BM38	ChiCTR1800018143 [49]	Lentivirus	RRMM (≥2 lines, n=22)	BCMA/CD38	Single	Phase 1 (recruiting)	ORR 87%; sCR 52%; MRD^-^ rate 87%; median PFS 17.2 months	CRS 87%, ≥ 3 grade 22%; neurotoxicity 0%
Anti-CD19 and anti-BCMA	ChiCTR-OIC-17011272 [50]	Lentivirus	RRMM (≥4 lines, n=21)	CD19/ BCMA	Combined	Phase 2 (recruiting)	ORR 95%; ≥ CR 57%; MRD^-^ rate 81%	CRS 90%, ≥ 3 grade 5%; neurotoxicity 10%
Anti-CD19 and anti-BCMA	NCT03455972 [51]	Lentivirus	RRMM (n=10)	CD19/BCMA	Combined	Phase 1/2 (recruiting)	ORR 100%; sCR 90%; MRD^-^ rate 70%; median PFS not reached	CRS 100%, ≥ 3 grade 0%; neurotoxicity 0%
CT053	NCT03975907 (Lummicar-1) [52]	Lentivirus	RRMM (≥3 lines, n=14)	BCMA	Single	Phase 1 (active, not recruiting)	ORR 100%; ≥ CR 42%; MRD^-^ rate 42%	CRS 92%, ≥ 3 grade 0%; ≥ 3 grade neurotoxicity 0%
CT053	NCT03915184 (Lummicar-2) [53]	Lentivirus	RRMM (≥3 lines, including anti-CD38 MoAb, n=14)	BCMA	Single	Phase 1b/2 (recruiting)	ORR 100%; ≥ CR 29%; MRD^-^ rate 92%	CRS 86%, ≥ 3 grade 0%; ≥ 3 grade neurotoxicity 0%
P-BCMA-101	NCT03288493 (PRIME) [54]	Transposon	RRMM (≥3 lines, n=43)	BCMA	Single	Phase 1/2 (terminated)	ORR 57%	CRS 17%, ≥ 3 grade 2%
Bb21217	NCT03274219 (CRB-402) [55]	Lentivirus	RRMM (≥3 lines, n=46)	BCMA	Single	Phase 1 (active, not recruiting)	ORR 55%; ≥ CR 18%	CRS 67%; neurotoxicity 22%
Allo-715	NCT04093596 (DL3 or DL4 group) [56]	Lentivirus	RRMM (≥3 lines, including anti-CD38 MoAb, n=26)	BCMA	Single	Phase 1 (recruiting)	ORR 62%; MRD^-^ rate 31%	-

**Abbreviations:** Ide-cel, Idecabagene vicleucel/bb2121; CAR-T, chimeric antigen receptor T cells; RRMM, relapsed/refractory multiple myeloma; MoAb, monoclonal antibody; MRD^-^, MRD negative; Cilta-cel, ciltacabtagene autoleucel; BCMA, B-cell maturation antigen; ORR, overall response rate; CR, complete response; MRD, minimal residual disease; PFS, median progression-free survival; CRS, cytokine release syndrome; sCR, stringent complete response.

**Table 2:** Technological prospecting of CAR-T therapy.

**Optimized Strategy**	**Specific Measures**	**Clinical Trial (Status)**
Overcome antigen escape	Develop non-BCMA-directed CAR-T products	NCT04499339 (phase 1/2a); NCT03672318 (phase 1, recruiting); NCT03464916 (completed); NCT04555551 (phase 1)
	Develop dual-target or multi-target CAR-T products	Check Table **1**
	Increase target antigen density	NCT03502577 (phase 1, suspended)
Prevent CAR-T cell exhaustion	Optimize the CAR-T structure	NCT03338972 (completed); NCT03070327 (active, no recruiting); NCT03093168 (unknown status); ChiCTR1800018137 (phase 1)
	Utilize memory T cells	NCT03288493 (phase 1/2, terminated)
	Inhibit exhaustion-related signals	NA
	Improve the anti-tumor activity of CAR-T cells	NCT03778346 (unknown status); NCT03602157 (phase 1, recruiting)
Overcome immunosuppressive microenvironment	Modulate the immune checkpoint of CAR-T cells	NCT04162119 (unknown status)
Improve the safety of CAR-T cell therapy	Control the toxicity of CAR-T cells	NCT03093168 (unknown status)

**Abbreviations:** MM, multiple myeloma; CAR-T, chimeric antigen receptor T cells; BCMA, B-cell maturation antigen; NA, not available.

## LIST OF ABBREVIATIONS

MM: Multiple Myeloma
PIs: Proteasome Inhibitors
IMIDs: Immunomodulatory Drugs
CAR-T: Chimeric Antigen Receptor T Cells
RRMM: Relapsed Refractory Multiple Myeloma
